# T cell phenotype switching in autoimmune disorders: Clinical significance of targeting metabolism

**DOI:** 10.1002/ctm2.898

**Published:** 2022-07-29

**Authors:** Matteo Barberis, Alejandra Rojas López

**Affiliations:** ^1^ Systems Biology, School of Biosciences and Medicine Faculty of Health and Medical Sciences University of Surrey Guildford UK; ^2^ Centre for Mathematical and Computational Biology, CMCB University of Surrey Guildford UK; ^3^ Synthetic Systems Biology and Nuclear Organization Swammerdam Institute for Life Sciences University of Amsterdam Amsterdam The Netherlands

**Keywords:** autoimmune disorders, computational modelling, metabolism, multi‐scale modelling, phenotype switching, Systems Biology, T cell phenotypes

## Abstract

Increasing efforts points to the understanding of how to maximize the capabilities of the adaptive immune system to fight against the development of immune and inflammatory disorders. Here we focus on the role of T cells as immune cells which subtype imbalance may lead to disease onset. Specifically, we propose that autoimmune disorders may develop as a consequence of a metabolic imbalance that modulates switching between T cell phenotypes. We highlight a Systems Biology strategy that integrates computational metabolic modelling with experimental data to investigate the metabolic requirements of T cell phenotypes, and to predict metabolic genes that may be targeted in autoimmune inflammatory diseases. Thus, we propose a new perspective of targeting T cell metabolism to modulate the immune response and prevent T cell phenotype imbalance, which may help to repurpose already existing drugs targeting metabolism for therapeutic treatment.

## Graphical abstract

An imbalance in T cell phenotype switching triggers autoimmune disorders. The imbalance may initiate during the regulation of any of the interconnected cellular components that modulate T cell biology: environmental cues, signaling pathways, cytokine‐mediated regulation, cell‐to‐cell communication, cell cycle, metabolism, and microbiome. Failure of the coordination among these responses may kickstart the process of anomalous Th cell behavior, with the resulting Th cell phenotype imbalance potentially resulting in a switch from a healthy state to a disease state, thereby in the onset of autoimmune/autoinflammatory disorders.

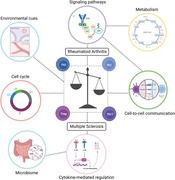

The immune system has developed multiple tolerance mechanisms to suppress the response against self‐antigens. These mechanisms can be operationally divided as central or peripheral depending on where they occur and aim to impair the activation of self‐reactive cells. The basic principle is to inactivate or promote apoptosis of a cell that exhibits a strong reaction to self‐antigens in central tissues, or a mild peripheral reactivity in absence of co‐stimulatory signals of a proper infection. This delicate balance is operated by the adaptive (or acquired) immunity, with specialized systemic cells—the lymphocytes T and B—and signalling cascades being devoted to eradicating non‐self‐antigens (pathogens) or damaged cells. However, when these immune cells are excessively zealous and react towards the host healthy cells, autoimmune disorders emerge. In other words, what makes the adaptative immune response immensely powerful, being able to generate strong and specific responses against non‐self‐pathogens, is also the reason behind the onset of autoimmune diseases. That is, the genetic mechanism that generates highly specific T cells or B cells—towards specific epitopes with a wide range of structures—always results in the production of cells reactive to cell's antigens. Therefore, tolerance mechanisms are in place to regulate homeostasis of immune cells, and their failure may lead to the escape of reactive cells that can initiate disease development.

Depending on what type of cell or tissue the anomalous response is developed against, a range of autoimmune diseases can be observed, including Multiple Sclerosis (reactivity against Schwann cells that form the myelin sheath that recovers neuronal axons), Rheumatoid Arthritis (immune response against the synovial membrane of cartilage and tendons), Systemic Lupus Erythematosus (production of antibodies against nuclear DNA), Crohn's disease (exacerbated inflammation of the digestive tract), Hashimoto's disease (reactivity against thyroid gland cells), Autoimmune Diabetes (immune response against the insulin‐producing β cells in the pancreas) and many others. These diseases have a dramatic impact on physiological functions, and understanding their development and pathogenesis is sought to develop increasingly better treatments that improve patients’ life quality. Particularly, as definitions of immune diseases are based on the reactivity to a type of self‐antigen, the molecular mechanisms underlying the pathogenesis and symptoms are not always the same. Thus, the prognosis and the response to different treatments are subject‐specific.^1^ Consequently, if the molecular and signalling basis for the development of an autoimmune disease varies from patient to patient, it becomes critical to tackle the common ground: (1) the specific involvement of different proliferating immune cell subtypes and their balance in disease onset, and (2) the molecular signals that may be responsible for switches from a physiological state to another, where an imbalance of the immune system may occur abruptly. Here, we highlight (1) T helper (Th) cells—immune response mediators that maximize the capabilities of the adaptive immune system—as the specific immune cells which subtype imbalance may lead to a switch from a physiological healthy state to a physiological (from a disorder's side) disease state^2^ (Figure 1), and (2) the metabolic pathways as non‐linear molecular signals modulating the Th phenotype, ultimately impacting on the quality of Th cell response.^3^, ^4^

**FIGURE 1:**
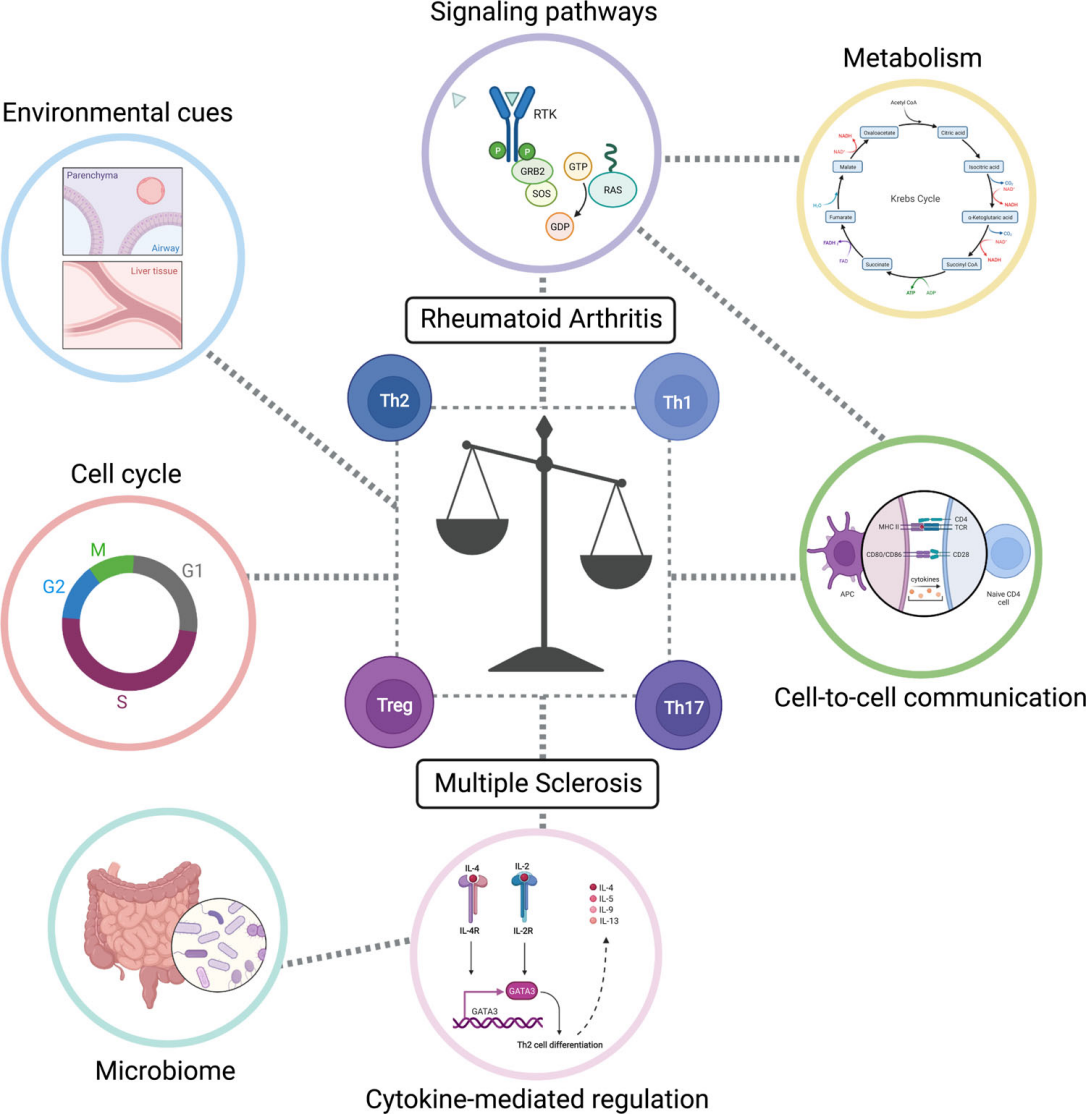
An imbalance in T cell phenotype switching triggers autoimmune disorders. The imbalance may initiate during the regulation of any of the interconnected cellular components that modulate T cell biology: *environmental cues* may be sensed by the cell, which signals are transmitted through multiple *signalling pathways*, *cytokine‐mediated regulation* and *cell‐to‐cell communication* between immune cells of different cell types; ultimately, signals may modulate the response of the *cell cycle* machinery, the *metabolism* and the *microbiome*. Failure of the coordination among these responses may kickstart the process of anomalous Th cell behavior, with the resulting Th cell phenotype imbalance potentially resulting in a switch from a healthy state to a disease state, thereby in the onset of autoimmune/autoinflammatory disorders. Created with and adapted from BioRender.com

Specifically, we propose that autoimmune disorders develop as consequence of a metabolic imbalance driving T cell phenotype switching. When considering the interplay in a cell among different layers of regulation, it is common to think hierarchically. First, the cell receives a signal stimulating proliferation; then, the signal reaches the gene expression machinery through molecular signalling cascades, which kickstarts the proliferation program (i.e. the cell cycle progression); finally, the signal is converted to a metabolic response to adapt to the demands of cell division, thereby of immune cell proliferation. However, at the system's level, all the molecular players can initiate a response by interacting with one another. A cell can change the gene expression program and cascade that information to other cells through signalling pathways or through both gene expression and molecular signalling. For example, the absence of oxygen, or a reduced flux through specific metabolic reactions can be a signal for the gene expression machinery to indicate that the energy conditions are not favourable for the cell to sustain proliferation.^2^

As it occurs for any cell types, Th cells have specific metabolic requirements that modulate their phenotype. Naïve Th cells before encountering an antigen rely mostly on fatty acid oxidation and oxidative phosphorylation to meet their energy requirements, whereas activated, functional Th cells rely more on glycolysis and increase glucose internalization through glucose transporters to support cell proliferation. An example of this modulation occurs for the transcription factor HIF1α, which regulates the anaerobic switch in the metabolism. In normal oxygen conditions, HIF1α is degraded, whereas in hypoxic conditions it promotes expression of glycolytic enzymes, thus increasing the flux of glycolysis and the consumption of glucose for both energy production and its conversion to biosynthetic precursors. Furthermore, HIF1α expression is pivotal for the differentiation of the Th17 phenotype while suppressing the Treg phenotype.^5^ The non‐linear axis between molecular signalling, gene expression, cell cycle and metabolism is key for cells to convert biochemical signals into specific functions. That is, to signal the stimulation by the CD28 ligand—the major co‐stimulation pathway for naïve T‐cell activation—, activation of the Akt pathway is central to modulate the expression of mTORC1 and HIF1α that promote cell growth and an increased glycolytic flux. Moreover, clonal expansion of Th cells involves a crosstalk of regulation of immune‐related genes with the cell cycle. In this context, cyclin‐dependent kinases (CDKs) phosphorylate targets related to the expression of immune genes, and inhibition of CDK activity through, as well as overexpression of, CDK inhibitors impairs T cell proliferation.^6^ Deficiency of the CDK inhibitor p21^Cip1^ results in the development of autoinflammatory diseases, while deficiency of the Cyclin D3/CDK6 complex activity leads to decreased immune cell numbers.

The relative strength of stimulation by CD28 and TCR (T cell receptor) receptors, and the subsequent signalling through activation of the mammalian target of rapamycin (mTOR) multiprotein complexes, mTORC1 (Complex 1) or mTORC2 (Complex 2), results in the differentiation of Th cells into Th1 or Th2 phenotypes, promoting activation of T‐bet (T‐box transcription factor TBX21 expressed in T cells) or GATA3 (GATA Binding Protein 3) transcription factors, respectively. The mTOR pathway is connected to the metabolic state of the cell that influences cell cycle and, in turn, gene expression dynamics. Yet, cell cycle molecules impact on gene expression and metabolic switches, and gene expression affects the timely activation of events in both metabolism and cell cycle. Deregulation of the highly non‐linear signalling that leads to T cell activation and differentiation can result in the onset of autoimmune/autoinflammatory disorders. Specifically, because the pathogenesis of these diseases is often dependent on the activation of Th cells, an imbalance among different Th phenotypes, thereby of their metabolic requirements, may be expected. Several studies highlight an imbalance between Th1/Th17 and Treg phenotypes in disease onset. The pleiotropic cytokine IL‐6 inhibits the function of TGFβ (transforming growth factor β), thus disfavouring the Treg phenotype—which would suppress adaptive T cell responses—to promote the Th17 phenotype. The latter correlates with the incidence and severity of immune diseases, such as Rheumatoid Arthritis^7^, ^8^ and Multiple Sclerosis^9^ (Figure 1). Similarly, increased Th1/Th17 with an enhanced glycolytic activity and decreased Treg populations are observed in patients affected by Psoriasis (autoimmune disease where T cells attack skin cells).^10^ Indeed, while the Treg phenotype relies on high fatty acid oxidation and oxidative phosphorylation fluxes, Th1/Th17 phenotypes rely on enhanced glycolysis and fatty acid synthesis.^11^, ^12^

Although the specific metabolic rewiring underlying T cell phenotype switching is largely unknown, recent studies that integrate computational and experimental analyses have investigated the metabolic requirements of T cell phenotypes. The combination of single‐cell RNA sequencing (single‐cell transcriptomics) with the network topology of the human metabolic map, analysed through metabolic modelling in the form of Flux Balance Analysis (FBA), has revealed polyamine metabolism as determinant of Th phenotypes differentiation.^13^ Here, flux distributions across metabolic reactions were predicted computationally and validated in Th17 cells in an experimental model of Multiple Sclerosis.^13^ In agreement with their pro‐inflammatory function in autoimmune inflammatory diseases, Th1 and Th2 phenotypes show an increased polyamine synthesis along with an increased glycolytic and tricarboxylic acid cycle (TCA) fluxes, compared to a very low flux of the polyamine pathway in non‐pathogenic Treg and Th17 phenotypes.^14^ In another study, by integrating transcriptomics and proteomics data, we have developed genome‐scale metabolic models of T cell phenotypes and, through FBA, we have identified the specific metabolic genes that may be targeted in autoimmune inflammatory diseases, among which Rheumatoid Arthritis and Multiple Sclerosis.^15^ By exploring target genes whose deletion would impact negatively (positively) the flux changes through metabolic reactions controlled by genes up‐regulated (down‐regulated) in diseases, a metabolic switch was unraveled for Th1 and Th2 phenotypes. While predicted targets for Multiple Sclerosis were identified in the glutaminolysis of Th2 cells (with enhanced reaction fluxes compared to Th1 cells), those for Rheumatoid Arthritis were identified in the glycolysis of Th1 cells (with enhanced reactions fluxes compared to Th2 cells).^15^ The Th1 phenotype and its effector cytokines are inferred to play a major role in the onset and development of Rheumatoid Arthritis^8^; strikingly, our model predictions restrict the complexity in the understanding of the disease by identifying specific metabolic pathways to be targeted in drug therapy. This bulk of evidence suggests that metabolism may be targeted to control autoimmune disorders by promoting the Treg phenotype over other effector phenotypes involved in the inflammation and progression of the diseases. Notably, ozone treatment ameliorates the symptoms in Multiple Sclerosis patients, by promoting mitochondrial metabolism and preventing the production of reactive oxygen species (ROS) thus acting as an antioxidant. Ozone treatment results in an increased frequency of Treg and in an increased expression of IL‐10, among other Treg‐related factors,^16^ which is an anti‐inflammatory cytokine exerting a protective role in autoimmunity through suppression of inflammatory cytokines. Of note, small molecules that induce IL‐10 production can be repurposed to promote IL‐10‐producing cells in vivo, providing an effective strategy of immune therapy.^17^

By analysing different experimental settings, such as –omics approaches (genomics, [single‐cell] transcriptomics, proteomics, metabolomics, etc.), through computational modelling, we and others aim to understand the molecular basis of the modulation of immune responses in health and disease. In particular, metabolic changes would reflect on the temporal activation of cell proliferation through specific gene expression changes. However, to untangle the difference between Th cell phenotypes, it is not enough to know which cell's receptors are expressed, or which cytokines are being produced. It is relevant to know the extent of their expression level or dosage^18^ along with the signalling and metabolic context. Therefore, to combine large datasets and derive molecular mechanisms specific for health or disease scenarios, it is indispensable to use a Systems Biology strategy that employs modelling tools to handle the complexity, variability and non‐linearity of biological interactions^19^ (Figure 2). To model aspects of the biology of autoimmune diseases is a challenge. Diverse mathematical and computational tools can be used depending on the type of available experimental data.^20^ For example, a map of protein–protein interactions can be modelled through Boolean modelling, in which each protein is represented as a node, and interactions between nodes are described by a set of logic rules. Instead, transcriptomics data that allow for mapping of the expressed molecules on the KEGG databases—to highlight the specific reactions involved—can be modelled through FBA, which explores the global response of the metabolism to perturbations. Conversely, if detailed information about a biochemical or enzymatic process is available, kinetic modelling may be used to propose a mechanism that would explain the observations and predict novel scenarios testable experimentally. An even bigger challenge is to integrate data from different levels of regulation, such as molecular interactions, enzymatic reactions, signalling cascades, metabolic pathways, different levels of gene expression regulation (from chromatin structure and transcription to epigenetics), cell cycle progression, cell to cell communication, and tissues structure, to rationalize organismal function. These complex functional modules, occurring at different time scales and in different subcellular compartments, may be integrated in multi‐scale models. These use different types of modelling formalisms to explain how non‐linear interactions within and among the modules work together to rationalise how the immune response occurs timely^21^ (Figure 2). To understand how disease scenarios such those emerging in autoimmune disorders develop, it is therefore crucial to investigate: (1) how the different regulatory layers integrate to carry out a healthy biological function, and (2) how a switch in the molecular context may explain why a pathway activation has different consequences in different T cell phenotypes for the emergence of an autoimmune disorder.

**FIGURE 2:**
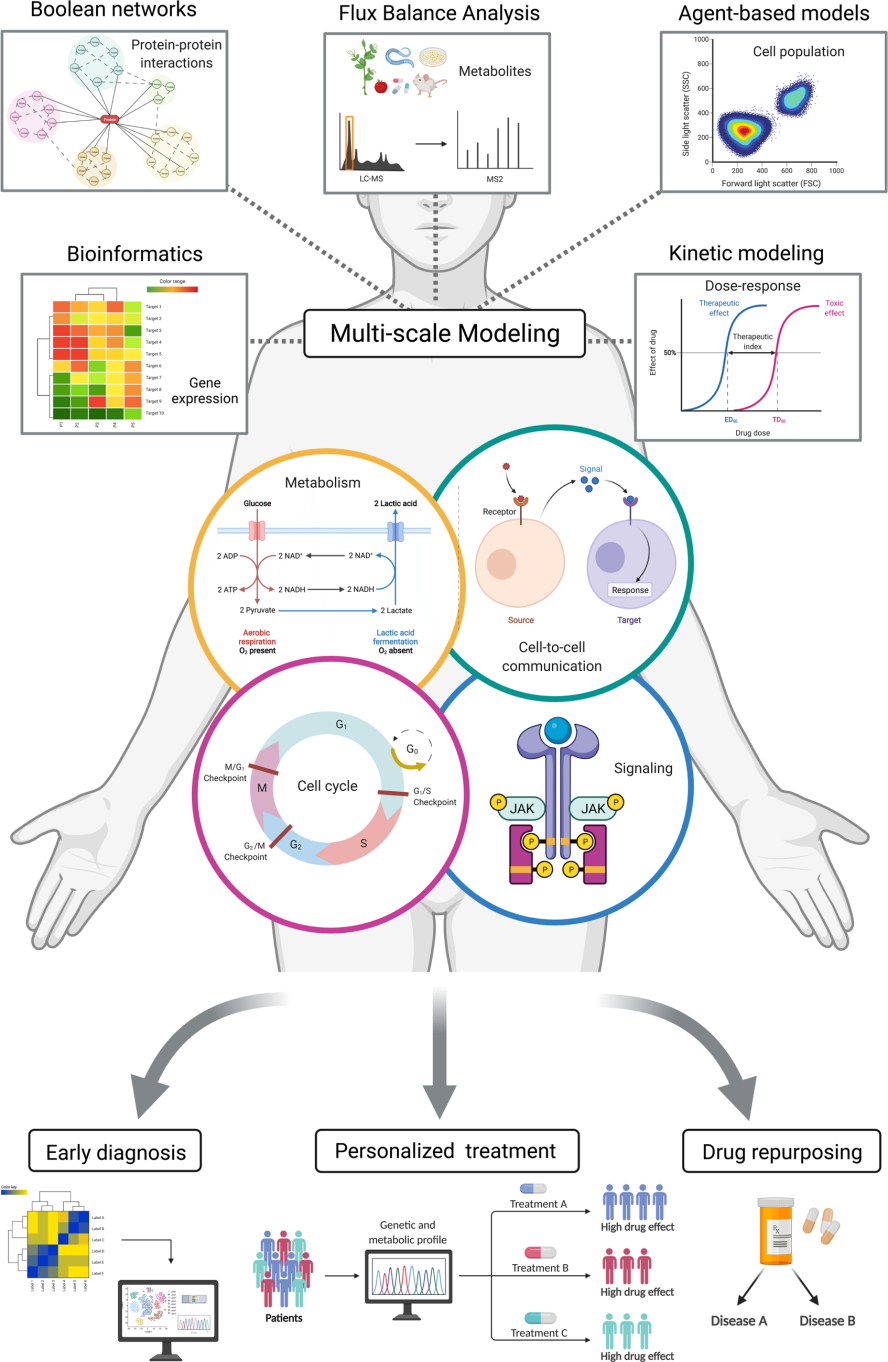
Integration of multiple sources of data and modelling formalisms in a multi‐scale view, to unravel the molecular mechanisms underlying the biology of T cells and their role in autoimmune disorders. (From top to bottom) Different formalisms are suitable depending on the type of available data: (i) Boolean models are practical to simulate the behavior of large networks of interacting molecules (e.g., protein–protein interactions); (ii) agent‐based models are useful when modelling cell populations or spatial regulation of molecular signals; (iii) kinetic modelling is suitable when handling detailed mechanistic information about a biochemical pathway/system; (iv) Flux Balance Analysis (FBA) is used to investigate the whole metabolism of a cell type if the information about the reactions taking place is available. In addition, (v) bioinformatic tools allow for the analysis of large, –omics data sets spanning, among others, the genome, the transcriptome, the proteome and the metabolome. Although different modelling approaches can be used to address specific Th cell‐related questions, integration of processes that occur at a different time (temporal) and spatial scales requires the combination of multiple formalisms in a multi‐scale modelling framework. The modelling formalisms may be used to investigate different aspects of T cell biology in health and disease scenarios. For example, metabolism, cell cycle regulation, gene expression through activation of multiple signalling cascades among which those activated by the cytokines environment, and cell‐to‐cell interactions, modulate T cell survival, proliferation and differentiation into multiple phenotypes. Ultimately, the specific Th/Treg phenotype populations, along with their secreted cytokines that regulate other immune cells, are responsible for the imbalance resulting in the onset and development of autoimmune/autoinflammatory disorders. Understanding how these aspects integrate shall improve the diagnosis and treatment of patients, through individual therapy. Specifically, the availability of information about patients’ metabolic and Th cell phenotype profiles will allow for the development of personalized therapies that match the former. Furthermore, the knowledge of metabolic markers that are related to autoimmune disease development will allow for an early diagnosis of the disease along with the prediction about the patient's prognosis and response to available treatments. Finally, due to the challenge that the development of new drugs poses, the understanding of common regulatory mechanisms among different autoimmune/autoinflammatory disorders opens opportunities for drug repurposing. Created with and adapted from BioRender.com

Altogether, metabolic pathways that are differentially expressed among T cell phenotypes may affect preferentially T cell types that are involved in disease development and progression. Thus, it may be possible to test whether: (1) known drugs that target these metabolic pathways are effective as treatments against autoimmune diseases, (2) modulating the T cell phenotypes ratio changes the temporal progression of the disease, and (3) modulating the T cell phenotypes ratio changes the response to a certain treatment. Zooming in, integration of metabolism, cell cycle and signalling pathways can provide further information on T cell development and phenotype switching, to understand immune responses and their deregulation. Thus, making a step from metabolism as ‘marker’ of a certain disorder to a framework for predicting the response to known treatments (or to test new treatments). In other words, targeting the T cell metabolism can modulate the immune response and prevent T cell imbalance.^22^ Therefore, the understanding of the metabolic ground of autoimmune disorders and the identification of pathways preferentially used by diseased cells can help to repurpose already existing drugs targeting metabolism for therapeutic treatment.^15^

## CONFLICT OF INTEREST

The authors declare no conflict of interest.

## FUNDING INFORMATION

This work was supported by the Systems Biology Grant of the University of Surrey to Matteo Barberis. Alejandra Rojas López was supported by the Systems Biology of Cell Cycle Control studentship of the Faculty of Health and Medical Sciences (FHMS) of the University of Surrey to Matteo Barberis.

## AUTHOR CONTRIBUTIONS

Matteo Barberis conceived and formulated the ideas and hypotheses, drawn the logic of the manuscript and figures, and wrote the manuscript. Alejandra Rojas López wrote the manuscript and helped with the initial drafting of the figures.
